# Increased Antibiotic Resistance of Methicillin-Resistant *Staphylococcus aureus* USA300 Δ*psm* Mutants and a Complementation Study of Δ*psm* Mutants Using Synthetic Phenol-Soluble Modulins

**DOI:** 10.4014/jmb.2007.07034

**Published:** 2020-10-08

**Authors:** Hun-Suk Song, Shashi Kant Bhatia, Tae-Rim Choi, Ranjit Gurav, Hyun Joong Kim, Sun Mi Lee, Sol Lee Park, Hye Soo Lee, Hwang-Soo Joo, Wooseong Kim, Seung-Oh Seo, Yung-Hun Yang

**Affiliations:** 1 Department of Biological Engineering, College of Engineering, Konkuk University, Seoul 05029, Republic of Korea; 2 Institute for Ubiquitous Information Technology and Applications (CBRU), Konkuk University, Seoul 1466, Republic of Korea; 3 Department of Biotechnology, College of Engineering, Duksung Women's University, Seoul 0169, Republic of Korea; 4 College of Pharmacy and Graduate School of Pharmaceutical Sciences, Ewha Womans University, Seoul 03760, Republic of Korea; 5 Department of Food Science and Nutrition, Catholic University of Korea, Bucheon 14662, Republic of Korea

**Keywords:** MRSA, phenol-soluble modulins, β-lactam antibiotic, biofilm, fatty acid, persister cell

## Abstract

Phenol-soluble modulins (PSMs) are responsible for regulating biofilm formation, persister cell formation, *pmtR* expression, host cell lysis, and anti-bacterial effects. To determine the effect of *psm* deletion on methicillin-resistant *Staphylococcus aureus*, we investigated *psm* deletion mutants including Δ*psmα*, Δ*psmβ*, and Δ*psmαβ*;. These mutants exhibited increased β-lactam antibiotic resistance to ampicillin and oxacillin that was shown to be caused by increased Nacetylmannosamine kinase (*nanK*) mRNA expression, which regulates persister cell formation, leading to changes in the pattern of phospholipid fatty acids resulting in increased anteiso-C_15:0_, and increased membrane hydrophobicity with the deletion of PSMs. When synthetic PSMs were applied to Δ*psmα* and Δ*psmβ* mutants, treatment of Δ*psmα* with PSMα1-4 and Δ*psmβ* with PSMβ1-2 restored the sensitivity to oxacillin and slightly reduced the biofilm formation. Addition of a single fragment showed that α1, α2, α3, and β2 had an inhibiting effect on biofilms in Δ*psmα*; however, β1 showed an enhancing effect on biofilms in Δ*psmβ*. This study demonstrates a possible reason for the increased antibiotic resistance in *psm* mutants and the effect of PSMs on biofilm formation.

## Introduction

Methicillin-resistant *Staphylococcus aureus* (MRSA) strains are regarded as major human pathogens [1]. These bacteria cause many diseases ranging from pneumonia to skin infections; most importantly, they have become resistant to antibiotics, resulting in problems during treatment [2]. MRSA are classified into two groups, *i.e.*, healthcare-associated MRSA (HA-MRSA) and community-associated MRSA (CA-MRSA), depending on the infection site [3]. CA-MRSA are different from HA-MRSA in that they generally contain Panton-Valentine leukocidin (PVL), which used to be the major relevant virulence factor of CA-MRSA skin and soft-tissue infections [4]. Furthermore, it has recently been determined that CA-MRSA express more phenol-soluble modulins (PSMs), and are therefore more virulent than HA-MRSA [5]. In other words, PSMs are significant major effectors of skin and soft-tissue infections, compared to PVL [6]. Along with PVL and alpha-hemolysin (Hla), PSMs are major virulence factors that are highly expressed in *Staphylococci* and CA-MRSA [7].

PSMs are a group of small, amphipathic peptides that have an α-helical structure, and there have been seven PSMs discovered, including PSM α1- α4, PSM β1-2, and PSMδ [8]. The general functions of PSMs include host cell lysis, biofilm formation, regulating persister cell formation, *pmtR* regulation and anti-bacterial effects [9]. *psm* genes are regulated by the *agr* system that positively regulates capsule formation, as well as extracellular protease and hemolysin production [10]. However, PSMs negatively regulate cell metabolism and the expression of biosynthetic genes [11]. To consolidate virulence in CA-MRSA, agr systems have been expressed to produce PSMs during the late exponential phase [12]. Recently, Δ*psm* mutants have produced thicker biofilms compared to those of the wild-type strain; it is unknown how PSMs influence physiological changes in MRSA [13].

In this study, the antibiotic resistance of the Δ*psm* mutants was compared to that of the LAC wild-type strain (USA300). Each PSM fragment and the whole peptide were supplemented to confirm the reversal of antibiotic susceptibility and elucidate the roles of each fragment. The expression profiles of N-acetylmannosamine kinase (*nanK*) and penicillin-binding protein 2a (encoded by *mecA*) were determined to investigate the regulatory function of PSMs related to antibiotic resistance. Membrane fatty acid profiling was performed to determine if the modified microenvironment was affecting the mechanism of antibiotic resistance. Additionally, membrane fluidity and cell surface hydrophobicity were compared to determine their relationship with antibiotic resistance.

## Materials and Methods

### Bacterial Strains, Media, and Culture Conditions

For cell preparation, the wild-type strain *S. aureus* USA300 (LAC, ATCC BAA1756) [14] and the mutant strains Δ*psm*α, Δ*psm*β, and Δ*psm*αβ [15] were cultured in tryptic soybean broth (TSB) agar and/or liquid broth. For pre-culture, a single colony of the strain from a TSB agar plate was used to inoculate 5 ml of TSB medium. Then, 1% (v/v) of the cell culture suspension was inoculated in a 96-well plate for the antibiotic resistance test, and cells were cultured overnight in a 37°C incubator without shaking, unless stated otherwise. PSMs were synthesized and prepared, as described previously [16].

### Analysis of Cell Growth and Biofilm Formation 

For analysis of cell and biofilm growth, a 96-well microplate reader was used to detect the optimal density at 595 nm (Tescan, Switzerland). Culture suspension (1%, v/v) from the pre-culture was inoculated into a 96-well plate and incubated for 24 h without shaking. Biofilm formation was analyzed using crystal violet, in accordance with previous protocols [17]. Briefly, the supernatant was discarded, and the biofilm was fixed with methanol and air-dried. Then, the biofilm was stained with 200 μl 0.2% crystal violet for 5 min, after which, the crystal violet was discarded, the plate was washed with distilled water, and air-dried. Finally, the biofilm was analyzed at 595 nm using a 96-well microplate reader (Tescan).

### Semi-Quantitative Reverse Transcriptase Polymerase Chain Reaction (RT-PCR)

Pre-culture was conducted using 5 ml of TSB, initiated using a single colony from an agar plate, in a shaking incubator at 37°C, overnight. Cells were cultured using 5 ml TSB with 1% inoculum in a shaking incubator at 37°C for 12 and 24 h to extract total RNA. Cells were harvested by centrifugation at 3,521 ×*g* for 20 min. Then, total RNA was prepared using the TRIzol Reagent and reverse transcription was performed using Superscript IV Reverse Transcriptase (Invitrogen Co., USA) to generate cDNA, as per the manufacturer’s instructions. Primers were designed using Primer express software v3.0.1 from Thermo Fisher Scientific (USA) (Table S1). These primers generated amplicons of 150 bp when comparing gene expression. Prior to semi-quantitative PCR, the cycle number was optimized to determine the saturated gene expression levels of DNA gyrase subunit B (*gyrB*, the endogenous control) for each template. After optimization, the optimal cycle number was set at 25 cycles to enable the further comparative analysis of gene expression. Semi-quantitative PCR was conducted using LA taq with GC buffer I (Takara Medical Co., Ltd.), using the methods in the manual.

### Fatty Acid Analysis 

Gas chromatography (GC)/mass spectrometry (MS) was used for the detection and quantification of fatty acids, in accordance with a previously described method, with a slight modification [18]. For methanolysis of fatty acids, approximately 10 mg of freeze-dried cells was weighed and placed in Teflon-stoppered glass vials. Then, 1 ml chloroform and 1 ml methanol/H_2_SO_4_ (85:15 v/v %) were added to the vials, after which they were incubated at 100°C for 2 h, cooled to room temperature, and then incubated on ice for 10 min. After adding 1 ml ice-cold water, the samples were thoroughly mixed by vortexing for 1 min, and then centrifuged at 3,521 ×*g*. The bottom organic phases were extracted using a pipette and moved to clean, borosilicate glass tubes, containing Na_2_SO_4_. GC/MS chromatography was performed using a Perkin Elmer Clarus 500 Gas Chromatograph that was connected to a Clarus 5Q8S Mass Spectrometer at 70 eV (m/z 50–550; source at 230°C and quadruple at 150°C) in the electrospray ionization mode with an Elite 5 ms capillary column (30 m × 0.25 mm i.d. × 0.25 mm film thickness; PerkinElmer, USA). The carrier gas, helium, was used at a flow rate of 1.0 ml/min. The inlet temperature was maintained at 300°C, and the oven temperature was programmed at an initial temperature of 150°C for 2 min before increasing to 300°C at a rate 4°C/min; the temperature was maintained for 20 min. The injection volume was 1 μl, with a split ratio of 50:1. The structural assignments were based on the interpretation of the mass spectrometric fragmentation and confirmed by comparison with the retention times and fragmentation patterns of the standards and spectral data that were obtained from the Wiley (http://www.palisade.com) and National Institute of Standards and Technology (http://www.nist.gov) online libraries. Methyl heneicosanoate (10 mg/ml; 10 μl) was used as an internal standard.

### Membrane Fluidity and Cell Surface Hydrophobicity (CSH) Tests

Membrane fluidity was measured as a fluorescence polarization or anisotropy value. Harvested cells were treated as per the protocol described [19]. Briefly, the samples were washed twice with phosphate-buffered saline, pH = 7.0, resuspended, and incubated at 37°C for 30 min with 1,6-diphenyl-1,3,5-hexatriene (DPH; Life Technologies, USA) at a concentration of 0.2 μM (0.2 mM stock solution in tetrahydrofuran). Fluorescence polarization values were determined using a SpectraMax 2 microplate reader (Molecular Devices; 360/40 nm excitation and 460/40 nm emission) using sterile black-bottom Nunclon delta surface 96-well plates. The excitation polarized filter was set in the vertical position. The emission polarized filter was set either in the vertical (*I*_VV_) or horizontal (*I*_VH_) position. The polarization value was calculated using the following formula:

(1) $$P=\frac{I_{\mathrm{VV}}-I_{\mathrm{VH}}G}{I_{\mathrm{VV}}+I_{\mathrm{VH}}G}$$

where G is the grating factor, assumed to be 1.

Cell surface hydrophobicity was estimated using the following method [20]. Cells grown in TSB medium were harvested by centrifugation (3,521 ×*g*, 20 min, 4°C) at the stationary phase of growth. The cells were suspended in cold phosphate buffer to an optical density at 595 nm (OD_595_) of 0.6. Aliquots of the suspension (3 ml) were transferred to two glass tubes. *n*-Octane (0.6 ml) was added to one tube (sample), but not to the other (control). Both suspensions were agitated vigorously for 2 min and then allowed to stand for 15 min to allow for separation into n-octane and saline layers.

## Results and Discussion

### Increased Resistance of *psm* Mutants to β-Lactam Antibiotics

**Fig. 1:**
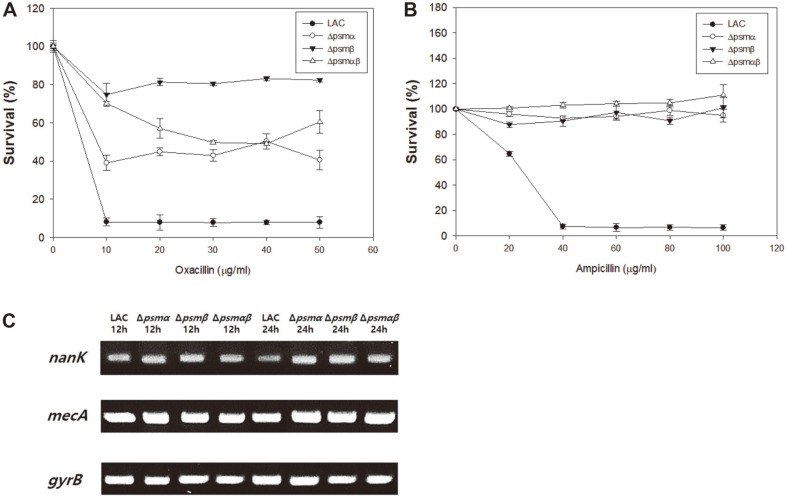
Investigation of antibiotic resistance in Δ*psm* mutants. (**A**) Identification of oxacillin resistance of Δ*psm* mutants. (**B**) Identification of ampicillin resistance of Δ*psm* mutants. (**C**) Semi-quantitative PCR of *nanK* and *mecA*. *gyrB* is used as an endogenous control. Error bars represent the standard deviation of three replicates.

PSMs are related to biofilm structure and dispersion in that they support the biofilm structure by generating an amyloid structure and are capable of disseminating the biofilm by virtue of their surfactant properties [21]. *psm* null mutants lose their surfactant ability and are therefore unable to disperse biofilms; this results in biofilm thickening, which might eventually culminate in increased antibiotic resistance [18]. Therefore, the antibiotic resistance of Δ*psm* mutants was compared to that of the wild-type MRSA LAC strain to determine how *psm* deletions would affect the antibiotic sensitivity. The wild-type MRSA strain also showed oxacillin and ampicillin resistance; however, the growth of wild type was inhibited in response to increased concentrations of oxacillin and ampicillin ( > 40 μg/ml) (Figs. 1A and 1B). In contrast, Δ*psm* mutant strains were able to grow, even in the presence of 50 μg/ml oxacillin and 100 μg/ml ampicillin; their growth was not inhibited with further increases in antibiotic concentration. To determine the minimum inhibitory concentration of oxacillin, the mutant strains were treated with increasing concentrations of oxacillin (Fig. S1). As Δ*psm* mutants showed a thicker biofilm compared to the wild-type LAC strain, leading to blockage of antibiotic access and generation of persister cells, which is relevant to *nanK* expression [15, 22], other reasons for increased resistance were examined.

As *mecA* is directly related to β-lactam antibiotic resistance, the expression of *mecA* in Δ*psm* mutants was investigated. The expression of *nanK*, which regulates the persister cell formation mentioned above, was also investigated (Fig. 1C). Mutations of *psm*α, *psm*β and *psm*αβ resulted in increased *nanK* expression, suggesting that all Δ*psm* mutants expressed more *nanK* than the wild-type strain and formed more persister cells. When the expression of *mecA* was compared, the difference between strains was not dramatic, suggesting the production of PBP2a protein was not an issue in the resistance of *psm* mutants.

### Analysis of Membrane Fatty Acid, Membrane Fluidity, and CSH of Δ*psm* Mutants

**Fig. 2:**
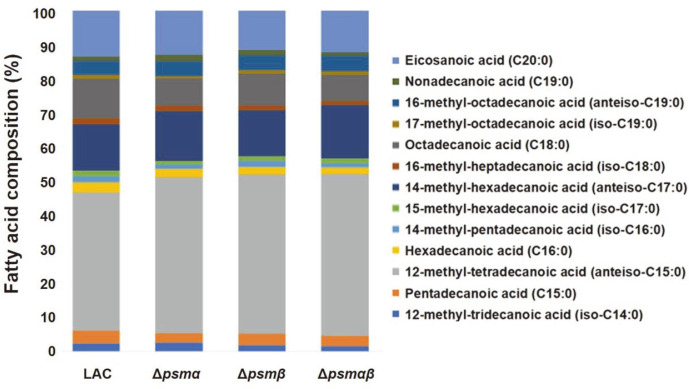
Membrane fatty acid profiles of the wild-type LAC strain and Δ*psm* mutants.

**Fig. 3:**
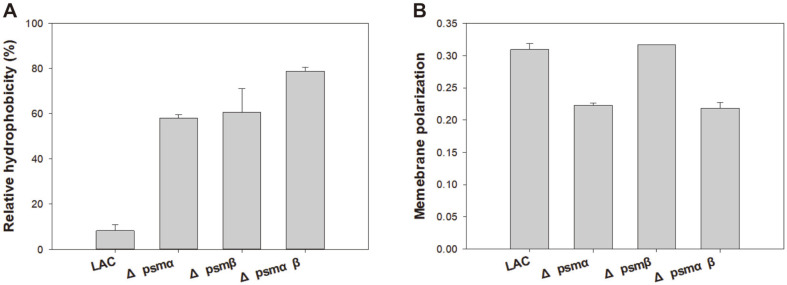
Analysis of cell surface hydrophobicity and membrane fluidity of the wild-type LAC strain and the Δ*psm* mutants. (**A**) Comparative analysis of cell surface hydrophobicity. (**B**) Comparative analysis of membrane fluidity. Error bars represent the standard deviation of three replicates.

The complexity of antibiotic resistance mechanisms can be attested by the fact that various factors affect the physiological changes in MRSA resulting in increased antibiotic resistance. Biofilm formation, fatty acid synthesis, CSH, membrane fluidity, and membrane permeability are the major factors responsible for antibiotic resistance [23-26]. To check if a compositional change in fatty acids is responsible for the development of antibiotic resistance, membrane fatty acids are analyzed using GC-MS [18]. Additionally, CSH and membrane fluidity were investigated as CSH is known to be related to biofilms, which result in lower exposure to the surroundings and a less fluid membrane in some isolated MRSA strains that are known to have higher resistance. Phospholipid fatty acid analysis (PLFA) showed that all Δ*psm* mutants contained higher anteiso-C_15:0_ content compared to that of the wild-type strain, suggesting that the higher anteiso-C_15:0_ content was related to increased antibiotic resistance (Fig. 2). With respect to CSH, Δ*psm* mutants seemed to be more hydrophobic, which might have affected the increased antibiotic resistance (Fig. 3A). However, no correlation was observed between membrane fluidity and antibiotic resistance (Fig. 3B). Considering the roles of the membrane, changes in PLFA and hydrophobicity could be evidence of increased antibiotic resistance.

### The Complex Effect of PSM Peptides on Antibiotic Susceptibility and Biofilm Formation 

**Fig. 4:**
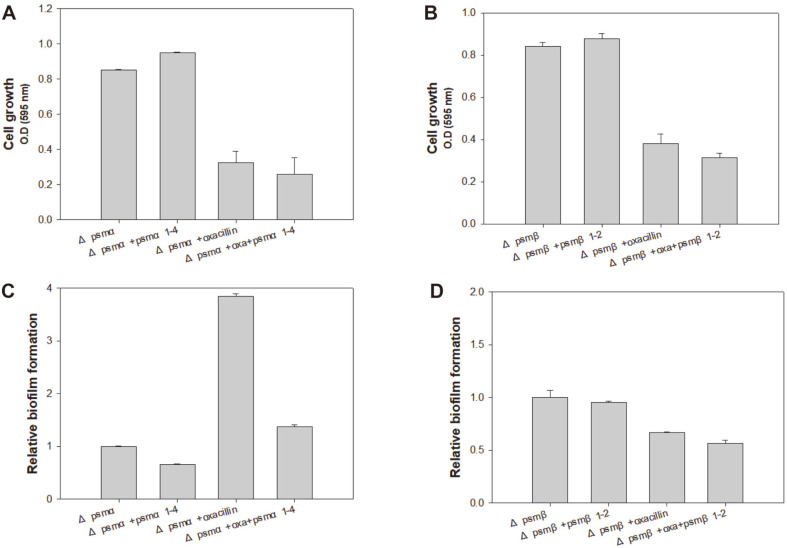
Reversed antibiotic susceptibility in Δ*psm* mutants with PSM peptide complementation. (**A, B**) Growth comparison of the wild-type LAC strain and the Δ*psm* mutants with PSM peptide complementation. (**C, D**) Biofilm formation of the wild-type LAC strain and Δ*psm* mutants with PSM peptide complementation. Error bars represent the standard deviation of three replicates.

As the loss of PSM peptides in MRSA resulted in increased antibiotic resistance, it was expected that the exogenous supply of PSM peptides to *psm* deletion mutants could possibly revert the antibiotic sensitivity, similar to that observed in the wild-type LAC strain. PSMs are composed of several fragment units and previous studies have demonstrated that they tend to have common and dissimilar features [27, 28]. The Δ*psm* mutants were independently exposed to all fragments of PSMα1-4 and PSMβ1-2. The treatment of Δ*psm* mutants with PSM fragments and the complementation of each deletion mutant with the fragments resulted in slightly increased growth in the absence of oxacillin; however, complementation reversed loss of susceptibility to antibiotics leading to decreased growth compared to the Δ*psm* mutants without PSMs (Figs. 4A and 4B), although it did not fully reverse antibiotic susceptibility to the levels observed in the wild-type strain. Wild-type and Δ*psm* mutant USA300 exhibited different biofilm formation patterns. In case of Δ*psm*α, oxacillin greatly increased the biofilm formation, which was clearly decreased by PSMα1-4 (Fig. 4C). The results obtained in Δ*psm*β were similar to those obtained in Δ*psm*α; however, the effect of PSMα1-4 was not as dramatic (Fig. 4D). Exogenous PSM supplementation revealed that PSMα1-4 decrease biofilms, while PSMβ1-2 have a mixed effect on biofilms, depending on the fragment.

### Effect of Each Peptide Fragment on Antibiotic Resistance and Biofilm Formation

**Fig. 5:**
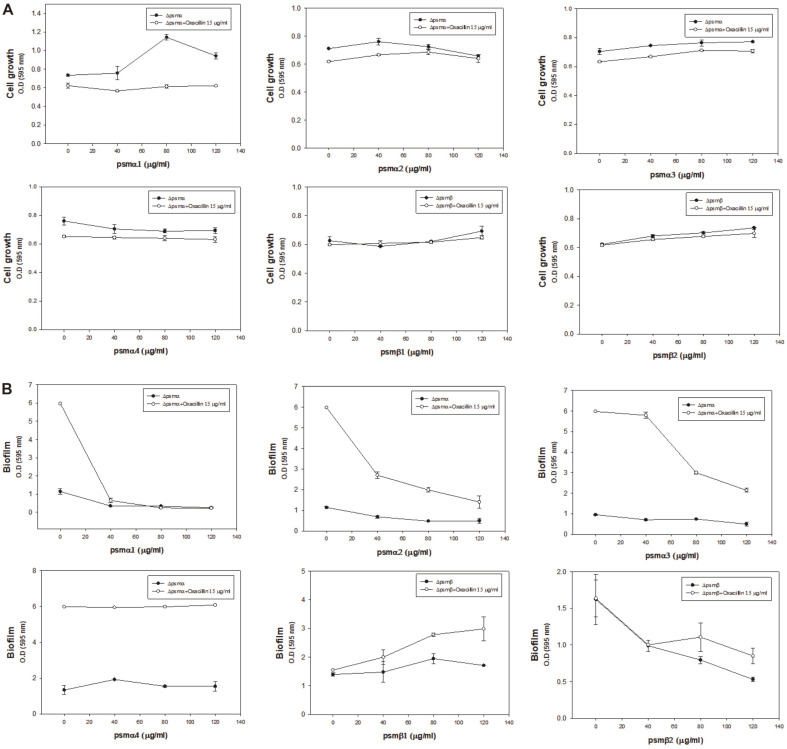
PSM fragment complementation into Δ*psm* mutants. (**A**) Growth of Δ*psm* mutants with PSM fragment complementation. (**B**) Biofilm formation of Δ*psm* mutants with PSM fragment complementation. Error bars represent the standard deviation of three replicates.

As PSMs are composed of several fragment units and they tend to have common and dissimilar features [27, 28], it was desirable to understand the effect of each PSM peptide. However, it is unknown which segment affects biofilm thickening and decreased antibiotic sensitivity. Therefore, six peptides from PSMα1- α4 and PSMβ1-2 were initially tested for complementation to determine if antibiotic resistance was reversed for Δ*psm*α and Δ*psm*β, respectively. However, single fragment complementation did not result in reversed susceptibility to oxacillin in both Δ*psm* mutants (Fig. 5A). Biofilm formation decreased upon complementation with PSMα1, PSMα2, and PSMα3 in Δ*psm*α, though PSMα4 did not exhibit any effect (Fig. 5B). Additionally, PSMβ1 showed an increased effect on biofilm formation by Δ*psm*β, but, PSMβ2 decreased biofilm formation by the Δ*psm*β mutant. These data explain the decrease in biofilm formation by Δ*psm*α exposed to a mixture of PSMα1-4 in Fig. 4C, and the mixed results in Δ*psm*β with PSMβ1-2 in Fig. 4D. Overall, among PSM peptides, PSMα1, PSMα2, PSMα3 and PSMβ2 decreased biofilm formation and PSMβ1 increased biofilm formation in a concentration-dependent manner. However, even with the decrease in the biofilm formation, the mutants did not show reversed susceptibility and remained antibiotic resistant.

## Conclusion

PSMs are involved in host cell lysis, anti-bacterial effects, control of biofilm formation and persister cell formation and are produced more in CA-MRSA than in HA-MRSA [28]. Without PSM, MRSA lose their ability to release cytoplasmic proteins and lipids into the supernatant [18, 29]. In addition, *psm* mutants produce thicker biofilms compared to the wild type [15]. Although important microscopy-based results have been identified, many roles of PSMs in the pathophysiology of MRSA remain unknown. This might be because PSMs were regarded as an offensive weapon to the human host and the link between an offensive weapon and the internal antibiotic resistance of MRSA seemed to be very weak. However, considering the survival strategy of MRSA, the loss of an offensive strategy need not necessarily result in weakness in the context of infection. Although MRSA might lose some virulence due to the absence of PSMs, there are other benefits, such as saving energy to produce PSMs or modifying their membrane structure. In addition, all MRSA strains have fewer PSMs than methicillin-sensitive *S. aureus* (MSSA), suggesting the complex role of PSMs in the context of virulence to the host and antibiotic resistance [30]. Thus, these findings describing the link between PSMs and oxacillin resistance demonstrate the sensitizing role of PSMs in MRSA.

To determine this, Δ*psm* mutants have been used to examine resistance to β-lactam antibiotics, as the Δ*psm* mutant strain is able to produce thicker biofilms and form persister cells [13, 31]. However, even with the surfactant effect of the PSM fragment leading to elimination of biofilms, Δ*psm* mutants did not become susceptible to β-lactam antibiotics. Thus, a biofilm is not the only factor affecting the mechanism underlying antibiotic resistance. With the use of deletion mutants *i.e.*, Δ*psm*, the expression of *nanK* and PLFA and hydrophobicity were changed. In addition to the fact that MRSA strains have lost their ability to release cytoplasmic proteins and lipids into the supernatant [29], a decrease in cell leakage and reduced fatty acids in the environment might facilitate membrane integrity followed by stable lipid raft formation, thereby increasing *nanK* expression to result in persister cell formation, leading to higher cell survival and cell wall hydrophobicity to help the cells attach more tightly to each other. This also may be a favorable situation for MRSA to oligomerize PBP2a into a functional conformation to increase antibiotic resistance [32]. Further investigation of fatty acids has shown that the anteiso-C_15:0_ proportion increased in the *psm* mutants. The increase in the acyl-chain length is responsible for the ordered state of the lipid bi-layer and aids the assembly of stable membrane microdomains, which further facilitate the oligomerization of PBP2a [18, 32]. In conclusion, PSMs clearly affect biofilm formation; however, they have more functions with respect to membrane composition and resistance-related genes that result in changes in the patterns of antibiotic resistance.

## Supplemental Material

Supplementary data for this paper are available on-line only at http://jmb.or.kr.
