# Supplementary figures and images for: Increased Antibiotic Resistance of Methicillin-Resistant Staphylococcus aureus USA300 Δpsm Mutants and a Complementation Study of Δpsm Mutants Using Synthetic Phenol-Soluble Modulins
Source: J Microbiol Biotechnol. 2020 Oct 8;31(1):115–22. doi: 10.4014/jmb.2007.07034 (PMC9705694; doi:10.4014/jmb.2007.07034)

Supplemental Fig. 1. Determination of oxacillin MIC level in  $\Delta psm$  mutants.

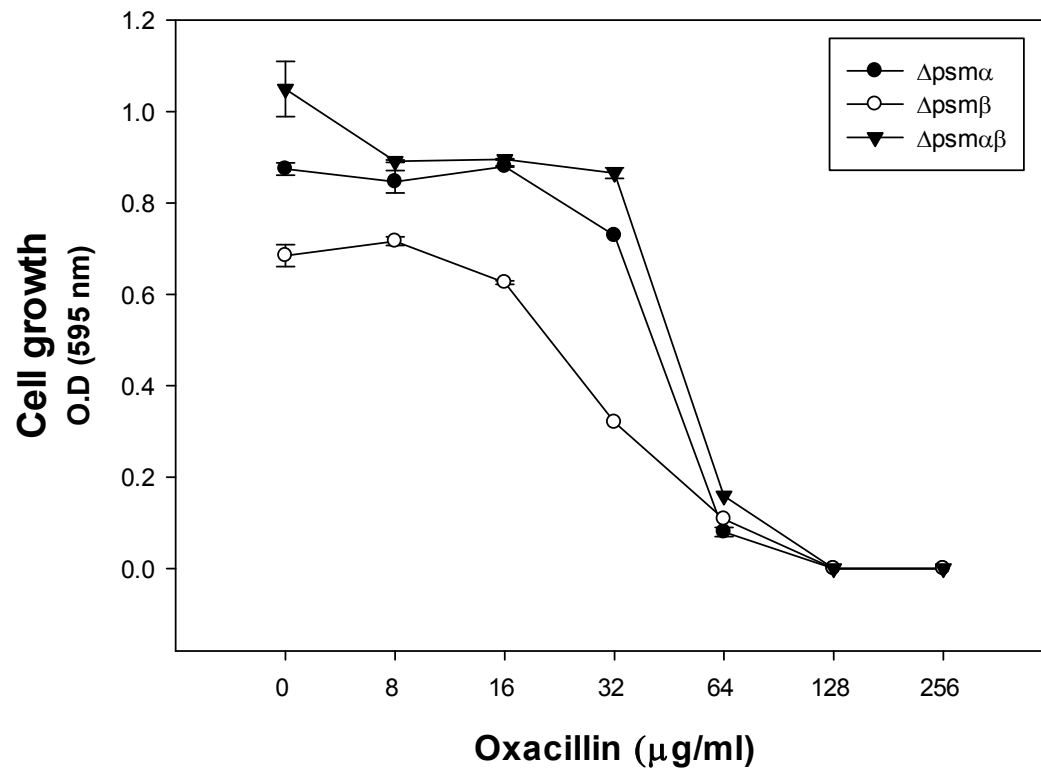

Supplement: Supplementary file 1 [file jmb-31-1-115-supple.pdf]
